# Probable IgG4-related sclerosing disease presenting as a gastric submucosal tumor with an intense tracer uptake on PET/CT: a case report

**DOI:** 10.1186/s40792-016-0161-6

**Published:** 2016-04-08

**Authors:** Ryota Otsuka, Masayuki Kano, Hideki Hayashi, Naoyuki Hanari, Hisashi Gunji, Koichi Hayano, Hisahiro Matsubara

**Affiliations:** Department of Frontier Surgery, Graduate School of Medicine, Chiba University, 1-8-1, Inohana, Chuo-ku, Chiba-shi, Chiba 260-8670 Japan

**Keywords:** IgG4-related sclerosing disease, Gastric submucosal tumor, Position emission tomography/computed tomography, Laparoscopic endoscopic cooperative surgery

## Abstract

A 44-year-old man consulted an internist because of abnormalities in an upper gastrointestinal series. It showed an elevated lesion with central depression in the greater curvature of the middle part of the stomach. Upper gastrointestinal endoscopy showed an elevated lesion with central depression, bridging hold, and no abnormalities of the gastric mucosa in the greater curvature of the middle part of the stomach. Endoscopic ultrasonography showed a submucosal tumor derived from the muscle layer of the stomach. Computed tomography showed a 22-mm tumor in the upper part of the stomach. Integrated position emission tomography/computed tomography (PET/CT) showed an intense tracer uptake by the tumor. Based on these findings, a gastrointestinal stromal tumor was suspected and laparoscopic endoscopic cooperative surgery was performed. A histopathological examination showed lymphoplasmacytic infiltration and fibrosis, and an immunohistochemical analysis showed the infiltration of IgG4-positive lymphoplasmacytic cells. The probable diagnosis was IgG4-related sclerosing disease of the stomach. We herein describe a rare case of probable IgG4-related sclerosing disease which presented as a gastric submucosal tumor. PET/CT is a useful imaging technique for the diagnosis and follow-up of this disease.

## Background

IgG4-related sclerosing disease is a systemic disease characterized by the extensive infiltration of IgG4-positive plasma cells and lymphocytes in various organs [1]. Although there are multiple recognized clinical manifestations of IgG4-related sclerosing disease, including sclerosing cholangitis, cholecystitis, retroperitoneal fibrosis, interstitial pneumonia, tubulointerstitial nephritis, and inflammatory pseudotumor [2], gastric lesions are rare. It has been reported that position emission tomography/computed tomography (PET/CT) is useful for the localized diagnosis of this disease [3].

We present a case of probable IgG4-related sclerosing disease presenting as a gastric submucosal tumor with an intense tracer uptake on PET/CT.

## Case presentation

**Fig. 1:**
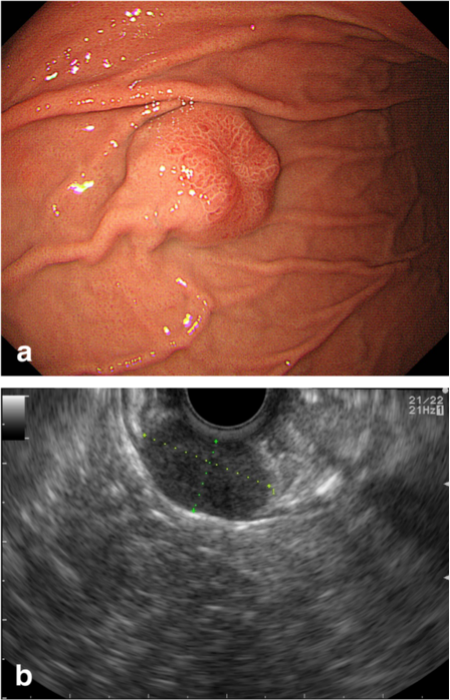
Upper gastrointestinal endoscopy shows the elevated lesion in the middle part of the stomach **(a)**. Endoscopic ultrasonography shows that the tumor is a submucosal tumor derived from the muscle layer of the stomach **(b)**

**Fig. 2:**
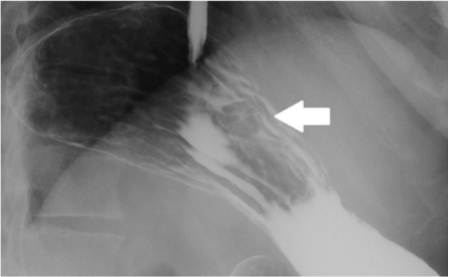
An upper gastrointestinal series shows an elevated lesion in the greater curvature of the middle part of the stomach

**Fig. 3:**
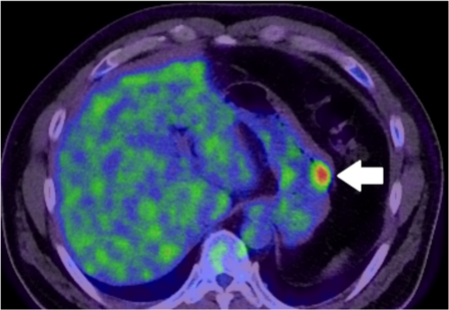
PET/CT shows an intense tracer uptake by the tumor in the upper part of the stomach

A 44-year-old man consulted an internist due to abnormalities on an upper gastrointestinal series. He had no relevant medical history. On examination, he appeared comfortable, with a temperature of 36.9 °C, blood pressure 152/97 mm Hg, and pulse 79 beats/min. The abdomen was soft, without tenderness or distension. The remainder of the examination was unremarkable. The laboratory data on admission showed a slightly decreased hemoglobin level of 13.3 g/dL, but the tumor marker levels (CEA, CA19-9) were almost within the normal limits. Upper gastrointestinal endoscopy showed an elevated lesion with central depression, bridging hold, and no abnormalities of the gastric mucosa in the greater curvature of the middle part of the stomach (Fig. 1a). Endoscopic ultrasonography showed that the well-demarcated, heterogeneous echo-poor mass originated from the muscle layer of the stomach (Fig. 1b). An upper gastrointestinal series showed an elevated lesion with a central depression in the greater curvature of the middle part of the stomach (Fig. 2). Computed tomography showed a 22-mm solid mass with a smooth margin and hypervascularity in the gastric wall at the upper part of the stomach. Integrated PET/CT showed an intense tracer uptake by the tumor (SUV_max_ = 6.8) (Fig. 3). Based on these findings, a gastrointestinal stromal tumor was suspected. Although the size of the tumor measured less than 5 cm and did not have clear malignant views, we judged this case to be a candidate for surgery in consideration of an intense tracer uptake by PET/CT. Laparoscopic endoscopic cooperative surgery was performed. First, the tumor location was confirmed at the greater curvature of the upper part of the stomach, after which the blood vessels in the excision area around the tumor were minimally ligated using an ultrasonically activated device. Next, endoscopic submucosal resection using endoscopic submucosal dissection (ESD) was performed around the tumor, and the seromuscular layer was intentionally perforated after three-quarters of the circumference of the excision had been finished. The tip of an ultrasonically activated device was then inserted into the perforation, and seromuscular dissection around the tumor was performed. After the tumor had been resected and removed, the incision line was closed using laparoscopic stapling devices. The operative time was 119 min, and blood loss was 5 mL.

**Fig. 4:**
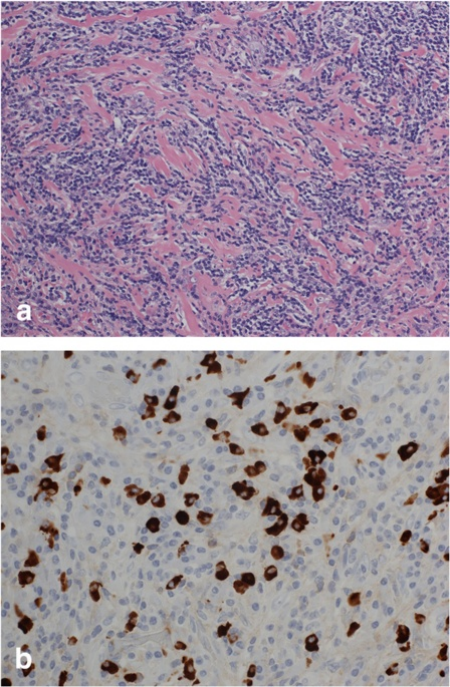
The histopathological findings show lymphoplasmacytic infiltration and fibrosis (×200) **(a)**. Immunohistochemistry findings show the infiltration of IgG4-positive lymphoplasmacytic cells (×400) **(b)**

The resected specimen showed a tumor measuring 20 × 18 mm in diameter, and its divided face was solid and poorly marginated. A histopathological examination showed lymphoplasmacytic infiltration and fibrosis (Fig. 4a), and an immunohistochemical analysis showed the infiltration of IgG4-positive lymphoplasmacytic cells (Fig. 4b). The patient was diagnosed with probable IgG4-related sclerosing disease of the stomach.

The patient made an uneventful recovery and was discharged on postoperative day 7. He was examined in a clinic 7 weeks after surgery and was found to have made a full recovery. Subsequent blood tests revealed that his serum IgG4 level was 98.1 mg/dL (reference range 4.8–105 mg/dL); however, the levels had not been examined prior to resection as an IgG4-related sclerosing disease had not been suspected.

### Discussion

The gastric manifestations of IgG4-related sclerosing disease are rare, and PET/CT is a useful imaging technique for their diagnosis and follow-up. The organ that is most commonly involved in IgG4-related sclerosing disease is the pancreas; such involvement is termed autoimmune pancreatitis (AIP) [1]. AIP is a specific type of pancreatitis that is thought to have an autoimmune etiology, and which typically shows the infiltration of IgG4-positive plasma cells and increased levels of serum IgG4. IgG4-positive plasma cells may involve not only the pancreas but also other organs, including the bile duct, gallbladder, salivary gland, thyroid gland, lungs, gastrointestinal tract, liver, retroperitoneum, kidney, prostate, and lymph nodes [4]. In 2003, Kamisawa et al. proposed a new clinicopathological entity, IgG4-related sclerosing disease, and suggested that AIP is a pancreatic lesion that reflects this systemic disease [5].

A diagnosis of IgG4-related disease is definitive in patients with the following findings: (1) organ enlargement, mass or nodular lesions, or organ dysfunction; (2) a serum IgG4 concentration of >135 mg/dL; and (3) histopathological findings of >10 IgG4 cells/HPF and an IgG4+/IgG+ cell ratio of >40 %. A diagnosis of IgG4-related disease is possible in patients who fulfill criteria (1) and (2), but with negative results on histopathology or without a histopathologic examination, whereas a diagnosis of IgG4-related disease is probable in patients with organ involvement (1) and in whom the histopathologic criteria are fulfilled, without an increase in serum IgG4 concentration (2) [4]. Our patient fulfilled criteria (1) and (3); thus, he was diagnosed with probable IgG4-related sclerosing disease. Although the patient’s serum IgG4 levels were normal after the resection of the submucosal tumor, they could have been elevated prior to surgery.

There are few reports of IgG4-related sclerosing disease presenting as a gastric lesion. Rollins et al. reported a gastric midbody mass which was histologically characterized as an IgG4-related autoimmune fibrosclerosing pseudotumor [6]. Fujita et al. reported an IgG4-related gastric ulcer without the main manifestation of autoimmune pancreatitis [7]. Only one case of IgG4-related gastric submucosal tumor was described by Murakawa et al. [8], in a patient who underwent laparoscopic endoscopic cooperative surgery. Thus, a gastric submucosal tumor of IgG4-related sclerosing disease is a rare manifestation.

Some reports have shown the usefulness of PET/CT imaging in IgG4-related sclerosing disease [3, 9]. PET/CT detects the various sclerosing lesions of the whole body; it is therefore effective for the diagnosis and follow-up of IgG4-related sclerosing disease. This disease is characterized by frequent temporal and spatial recurrence and requires a long-term follow-up. A continuous whole body search by PET/CT is therefore useful for IgG4-related sclerosing disease patients.

As corticosteroid therapy is effective in IgG4-related sclerosing disease, it may be considered the first treatment option. However, a therapeutic diagnosis with corticosteroid dosage is not recommended when differentiation with a malignant tumor is difficult [4]. Therefore, low invasive surgery such as LECS may be an effective therapy option for patients who are diagnosed with IgG4-related sclerosing disease presenting as a gastric submucosal tumor, such as the present case, in consideration of the side effects of corticosteroid dosages.

IgG4-related sclerosing disease is a relatively new disease concept, and its prognosis has not been well described. Additional studies of its long-term prognosis are needed.

## Conclusions

In summary, we herein described a rare case of probable IgG4-related sclerosing disease presenting as a gastric submucosal tumor. PET/CT is a useful imaging technique for the diagnosis and follow-up of this disease.

## Consent

Written informed consent was obtained from the patient for publication of this case report and any accompanying images. A copy of the written consent is available for review by the Editor-in-Chief of this journal.
